# Morphogenesis of Canine Chiari Malformation and Secondary Syringomyelia: Disorders of Cerebrospinal Fluid Circulation

**DOI:** 10.3389/fvets.2018.00171

**Published:** 2018-07-27

**Authors:** Susan P. Knowler, Gabriel L. Galea, Clare Rusbridge

**Affiliations:** ^1^Department of Veterinary Medicine, Faculty of Health and Medical Sciences, University of Surrey, Guildford, United Kingdom; ^2^Developmental Biology of Birth Defects, Great Ormond Street Institute of Child Health, University College London, London,, United Kingdom; ^3^Fitzpatrick Referrals Veterinary Specialist Hospital, Surrey, United Kingdom

**Keywords:** Arnold Chiari malformation, occipital hypoplasia, craniocervical junction, craniovertebral junction (CVJ), cerebrospinal fluid (CSF), brachycephaly, caudal occipital malformation syndrome (COMS)

## Abstract

Chiari-like Malformation (CM) and secondary syringomyelia (SM), as well as their analogous human conditions, is a complex developmental condition associated with pain and accompanying welfare concerns. CM/SM is diagnosed ever more frequently, thanks in part to the increased availability of magnetic resonance imaging in veterinary medicine. Research over the last two decades has focused primarily on its pathophysiology relating to overcrowding of the cranial caudal fossa. More recent characterizations of CM/SM include brachycephaly with osseous reduction and neural parenchymal displacement involving the entire brain and craniocervical junction to include rostral flattening, olfactory bulb rotation, increased height of the cranium, reduced cranial base with spheno-occipital synchondrosis angulation, reduced supraoccipital and interparietal crest and rostral displacement of the axis and atlas with increased odontoid angulation. The most shared manifestation of CM is the development of fluid-filled pockets (syrinx, syringes) in the spinal cord that can be readily quantified. Dogs with symptomatic CM without SM have a reduced basioccipital bone, compensatory increased cranial fossa height with displaced parenchyma whereby the cerebellum is invaginated beneath the occipital lobes but without compromising cerebrospinal fluid channels enough to cause SM. Thus, broadly defined, CM might be described as any distortion of the skull and craniocervical junction which compromises the neural parenchyma and cerebrospinal fluid circulation causing pain and/or SM. The etiology of CM is multifactorial, potentially including genetically-influenced, breed-specific abnormalities in both skeletal and neural components. Since causation between specific morphologic changes and SM or clinical signs is unproven, CM might be more appropriately considered as a brachycephalic obstructive CSF channel syndrome (BOCCS) rather than a single malformation. Understanding the normal development of the brain, skull and craniocervical junction is fundamental to identifying deviations which predispose to CM/SM. Here we review its anatomical, embryological, bio-mechanical, and genetic underpinnings to update the profession's understanding of this condition and meaningfully inform future research to diminish its welfare impact.

## Introduction

Chiari-like malformation (CM), and secondary syringomyelia (SM), is a painful inherited disorder common in brachycephalic toy breed dogs (1–5). CM/SM was highlighted as a substantial welfare concern by the Companion Animal Welfare Council (6, 7) over a decade ago. Since then, improved knowledge and understanding of the clinical and behavioral signs of the condition have increased the public and veterinary profession's awareness of its prevalence and the need to differentiate clinical CM/SM from normal non-pathogenic anatomical variations. SM is a disorder of cerebrospinal fluid (CSF) circulation and, in this review, confined to its dependent relationship to CM (8, 9). However SM can be asymptomatic and in the Cavalier King Charles Spaniel (CKCS) has a high prevalence (2, 3, 10). CM alone can be symptomatic (11) and any definition of CM should therefore take account of pathological abnormalities that not only disrupt CSF flow in the spinal canal with resultant syrinx formation but also cranial aberrations which can result in clinical and behavioral signs of pain. Classical clinical signs of SM in CKCS include phantom scratching which is a tendency to scratch towards one shoulder or neck region without skin contact (12) and shown to be related to a mid-cervical syrinx with a transverse width greater than 4 mm and/or extension to the region of the superficial dorsal horn (13, 14). Dogs with symptomatic SM may also present with neurological deficits such as scoliosis, weakness and proprioceptive deficits which reflect spinal cord damage from the expanding cavity. Syrinx independent signs include vocalization, scratching or rubbing of the facial region or ears, aversion to touch, spinal pain, exercise intolerance, refusal /difficulty jumping stairs, and sleep disruption are more likely to reflect CM (assuming other causes of this signs ruled out) (13, 15). To compound the complexity, clinical signs can be intermittent, have other possible causes or overlooked by owners. Many of the signs listed have been described and attributed to CM/SM in other breeds (16–20).

Both terminology and definitions of canine CM and SM are not only variable but, at times, controversial (21). Terms have included: Arnold Chiari malformation as with the human analog; occipital hypoplasia (OH) based on the reduced development of the basi and supraoccipital bone (22); and caudal occipital malformation syndrome (COMS) (23). Such names have resulted in confusion and in 2006 an international veterinary working group, invited by the UK Cavalier Club, were briefed to agree a name in order to dispel confusion (24). The suffix “like” was added to the human condition Chiari-I malformation because dogs do not have cerebellar tonsils and the severity of the condition is not dependent on the size of the cerebellar herniation (25). Although the working group agreed the acronym as CM/SM for Chiari-like malformation and secondary syringomyelia, CLM has been used as an acronym for CM in some research articles to distinguish it from the corresponding human condition.

The canine definition of CM has evolved over the last decade reflecting an increased understanding of its pathogenesis, and is no longer considered simply a reduced caudal fossa with an impacted and/or herniated cerebellum through the foramen magnum which compromises CSF channels (4, 8, 26, 27). In 2011, the British Veterinary Association (BVA)/Kennel Club (KC), as part of the CM/SM health screening programme, define 3 grades for CM (CM0-2) whereby the cerebellar morphology is assessed as a measure of overcrowding of the caudal fossa: the cerebellum in CM0 has a rounded shape with a high intensity signal on T2-weighted images consistent with CSF between the caudal cerebellar vermis and the foramen magnum; CM1 the cerebellum does not have a rounded shape, i.e., there is indentation by the supraoccipital bone, but there is a signal consistent with CSF between the caudal vermis and the foramen magnum, and CM2 the cerebellar vermis is impacted into or herniated through the foramen magnum. This historic grading system defined by the cerebellum does not relate to the severity of SM nor take account of dogs without CM but with SM (10, 28, 29). For example, a familial group of Griffon Bruxellois, with a rounded cerebellum (CM0), had ventriculomegaly, heighted cranial fossa and SM indicating disruption of CSF flow (29). Similarly, the description of Chiari malformation in humans has morphed over time and includes CM Type 0 with normally placed cerebellar tonsils (21, 30). The human classification of SM has also changed over time (25, 31) with advanced imaging techniques applied to CSF circulation and it is possible that further phenotypic variations for both SM and CM may be revealed.

**Figure 1:**
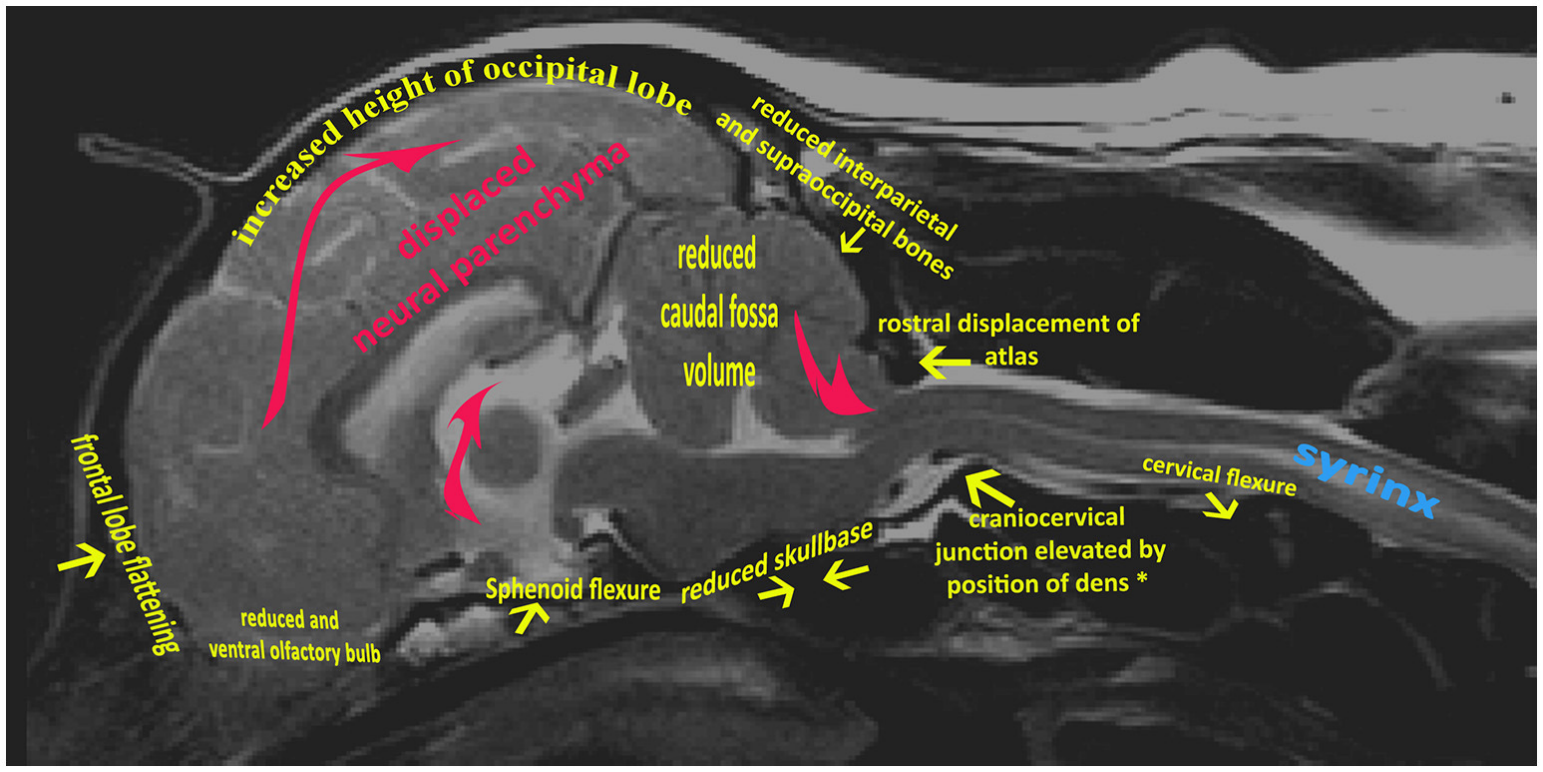
Mid-sagittal T2-weighted image of brain and cervical spinal cord of CKCS with CM and SM. Yellow arrows indicate generalized skeletal variances from normal CKCS skull and cervical junction. Red arrows indicate displaced neural parenchyma.

**Figure 2:**
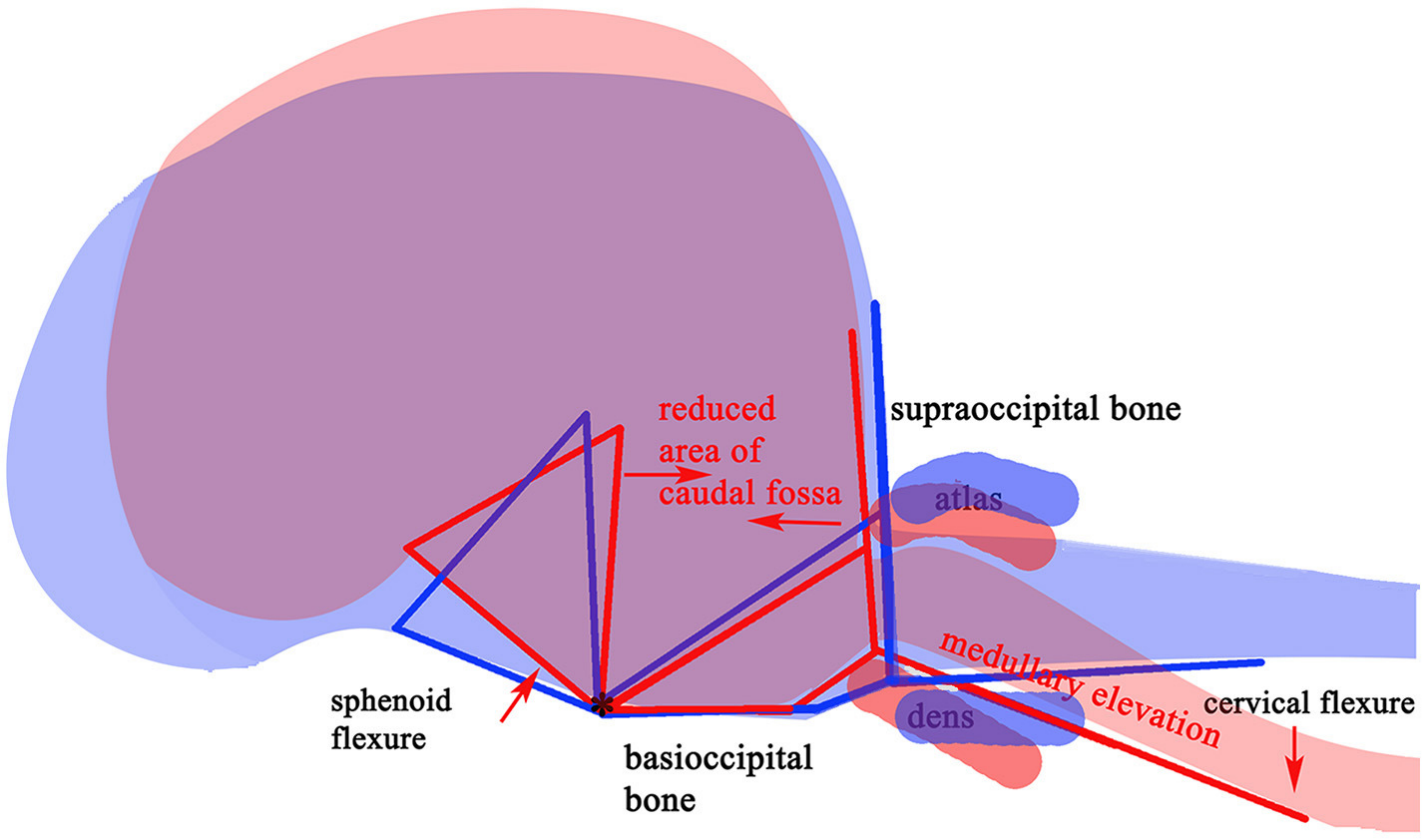
Schematic framework of selected CM traits (red lines) and “normal” traits (blue lines) to illustrate underlying anatomical differences in the caudal cranium and cranial cervical vertebrae. The red (SM affected) lines are superimposed on those for representing normal (blue) and aligned along the skull base at fixed point “*”, the dorsum of spheno-occipital synchondrosis. The basisphenoid, possibly the presphenoid, is flexed dorso-caudally (“sphenoid flexure”). The cranial cervical vertebrae are more rostral, with the odontoid process, angled ventrally with medullary elevation (“cervical flexure”).

The variable association of cerebellar herniation and craniocervical abnormalities with the occurrence and severity of SM, described above, implies involvement of additional anatomical abnormalities associated with CM. Indeed, recent characterization of CM/SM embraces the entire brain and not just the caudal cranial fossa and craniocervical junction (bony structures surrounding the junction between the medulla oblongata and spinal cord) (18, 27). The complexity of the disorder is illustrated on a mid-sagittal T2-weighted image of the brain of a CKCS with CM and SM highlighting the key anatomical components (Figure 1). The relationship between the significant traits associated with the cranial caudal fossa has been summarized in Figure 2. These include (i) shortening of the basicranium, (ii) spheno-occipital angulation, (iii) reduced occipital crest and supraoccipital, (iv) rostral displacement of the axis and atlas with increased odontoid angulation, (v) displacement of neural parenchyma with compensatory increase in cranial height, (vi) cerebellar herniation (11, 32). CKCS may have one or more of the features which predispose to SM which are typified by two scenarios depending on whether brachycephalic anomalies predominates over craniocervical deformities (32). Since the etiology of the syndrome has yet to be fully elucidated, a broad definition of CM, supported in this review, is **a malformation of the skull and craniocervical junction which compromises the neural parenchyma to cause pain and/or disrupt CSF circulation which can result in SM**. A diagnostic challenge is recognizing the combination and extent of the abnormalities associated with CM and SM. Histopathological aspects of CM/SM in the spinal cord (33) and their relationship with phantom scratching have been investigated (14), but further research is required to establish the pathophysiology of the pain associated with CM.

## Anatomical abnormalities in dogs with CM and secondary SM

A considerable body of research has been undertaken worldwide to understand the relationship/s between CM and SM in an attempt to elucidate the pathogenesis of SM in the dog. Attention has focused on secondary SM rather than CM. Various morphometric studies measured alterations in volume, and/or linear dimensions and/or angles of the cranium and craniocervical junction of dogs with and without CM/SM. The conclusions of key studies summarized below reflect the depth and breadth of investigations rather than a comprehensive literature review on the morphometrics of CM/SM:

- Caudal fossa was reduced in dogs with SM (34–37).
- Cerebellar volume was greater in CKCS with CM/SM (38) and abnormal in dogs with CM/SM (37, 39, 40) compared to normal controls.
- There was no correlation between severity of cerebellar herniation and neurological signs (39).
- There was a mismatch or rearrangement of neural parenchyma with SM-affected dogs (29, 35, 41, 42).
- SM-affected young dogs developed more severe clinical signs and syringes that enlarge over time (43–45).
- There was no difference in volumetric measurement of the neural parenchyma with changes in head position during MRI but there was a significant difference between the cerebellar herniation and CSF space between the cerebellum and brainstem which was larger in the flexed position (46).
- Venous sinus volume in the caudal cranial fossa is reduced in CKCS with CM/SM compared to dogs with CM (47) and the skull base is reduced, with narrowing of the jugular foramina which theoretically could increase intracranial venous pressure and impair CSF absorption (18, 48).
- CSF flow was impeded in dogs with CM/SM (26) and this could lead to a mismatch in the timing of arterial and CSF pulse waves predisposing to SM (49).
- Maximum syrinx width was the strongest predictor of pain, phantom scratching and scoliosis in dogs with SM. Both pain and syrinx size were positively correlated with syrinxes located in the dorsal half of the spinal cord (50, 51).
- Craniocervical junction abnormalities did not predict development or worsening clinical signs of SM but may contribute to them. These included atlantooccipital overlapping (52), atlantoaxial bands (53), and medullary elevation (54).
- The presence of a CSF flow void in the mesencephalic aqueduct associated with ventricular enlargement and SM in dogs suggests CSF turburlence and possibly reduced intracranial compliance (55).

It is not surprising that any incongruities between the capacity of the skull and brain size is considered a disorder associated with brachycephaly and miniaturization (Toy dog breeds). However, the extent to which these two risk factors contribute to the morphological abnormalities associated with CM and secondary SM has not been fully investigated. Toy breed dogs that have been reported with SM secondary to CM include Cavalier King Charles spaniels (CKCS), King Charles spaniels, Griffon Bruxellois, Affenpinschers, Yorkshire terriers, French bulldogs, Havanese, Chihuahuas, Pomeranians, Boston terriers, Maltese dogs, Yorkshire terrier, Papillons, miniature Dachshunds, Shih Tzu, Bichon Frisé and several cross breeds (23, 56). Despite considerable variation in skull shape and body weight, these breeds are accepted as being small in size but not all are recognized in the dog world as brachycephalic with obviously shortened craniofacial bones.

### Brachycephaly

Brachycephalic dogs are increasingly popular (57) and unfortunately also increasing are the health problems associated with this conformational change (58). Brachycephaly comes from the *Greek* ‘brakhus' meaning ‘short' and ‘cephalus' Latinized form of the *Greek* Kεϕαλoς (Kephalos), derived from κεϕαλη (kephale) meaning ‘head'. It is classically associated with airway compromise as extreme brachycephalic breeds have facial skeleton foreshortening due to maxillary hypogenesis often with airorhynchy (retroflexion on the neurocranial axis). This results in conformation changes to the airways which are associated with breathing problems. Affected dogs also have brain changes including ventral olfactory bulb rotation, and virtual abolition of the frontal sinuses (59–63). However brachycephaly, as defined by cephalic or cranial index, occurs without facial foreshortening in the dog. Cephalic index is an anthropometric parameter utilizing head length and breadth which is used to categorize human head shape and in the investigation of disease states such as craniosynostosis, hydrocephalous and microcephaly (64) which have increased cephalic index. It is calculated as the ratio of the maximum width of the human fetal head × 100 divided by its maximum length (65). This was modified by Evans (66) to classify dog heads into dolichocephalic, mesaticephalic and brachycephalic. The width of the skull was defined as the widest interzygomatic arch distance (66, 67). By comparison craniofacial index (the muzzle length divided by the cranial length) is used to assess facial foreshortening in extreme brachycephalic dogs (59). Increased cranial index has been demonstrated as a risk factor for SM (68) and ventricular dilatation (69), supporting the observation that brachycephaly affects the CSF channels.

Theoretically, bony and soft tissue changes associated with brachycephaly may impact on the CSF drainage system predisposing to ventriculomegaly and raised intracranial pressure (9, 70). Basichondrocranium anomalies (the cartilaginous parts of an embryonic cranium) have been associated with stenosis of the jugular foramen and raised intracranial pressure in humans (71, 72). Jugular foramen stenosis in CKCS dogs has been described (48). Reduction and rotation of the olfactory bulb is also associated with brachycephaly (60) and may have an impact on CSF drainage. The olfactory CSF conduit is an evolutionary ancient system whereby CSF drains though the interstitial space from the medial temporal lobe along the lateral olfactory stria through the olfactory trigone down the olfactory tract to the olfactory bulb (73–75). The CSF percolates through the cribriform plate of the ethmoid bone to the nasal submucosa and nasal lymphatic system, and CSF drainage amenability and any aberrations predispose to ventriculomegaly, raised intracranial pressure (76–78) and SM (9, 79).Thus brachycephaly-related distortions of intra-cranial dimensions and impaired CSF drainage may explain the etiology of independent symptomatic CM (11), and be an additional pathogenic factor for the development of SM. The phenotypes of brachycephaly have considerable variation between dog breeds and consequently Brachycephalic obstructive airway syndrome (BOAS) also has variation between individual breeds and presents with different phenotypes and severity, for example, nostril obstruction in Pugs, French Bulldogs and Bulldogs (80). In these respects CM/SM can be considered comparable to BOAS i.e., a Brachycephalic obstructive CSF channel syndrome (BOCCS). Similarly to BOAS and the airway, BOCCS can be considered any distortion of the skull and/or craniocervical junction that compromises the neural parenchyma and CSF circulation causing pain and/or SM with variation between breeds and individuals.

### Craniocervical junction abnormalities

The craniocervical junction or craniovertebral junction (CVJ) plays a crucial role in upholding circulation of the CSF (81). Mechanically CVJ consists of a central pivot (basioccipital bone, dens and axis) and two rings (foramen magnum and atlas) (82) and any alteration in the one or more of these components has the potential to affect CSF flow. For example, osseous abnormalities of the reduced caudal cranial fossa and craniocervical junction are thought to predispose to increased hydrostatic differentials between the spinal cord and the subarachnoid space (83, 84). Alterations in conformation affect CSF dynamics, as revealed by studies using cardiac-gated cine balanced fast field echo (bFEE) MRI (26, 49). SM affected CKCS have reduced venous sinus volume with parenchymal “overcrowding” of the caudal cranial fossa suggesting a role for reduced venous drainage in the pathophysiology of SM (47).

In the toy dog breeds there exists a number of craniocervical junction abnormalities which have been identified as risk factors for SM (85). They include atlanto-occipital overlapping, (37, 86) which is similar to basilar invagination (BI) in humans (87) and defined as invagination of the odontoid process into the foramen magnum with ventral brainstem compression i.e., a more severe malformation (88). Less common canine craniocervical junction anomalies associated with CM include atlantoaxial subluxation (20, 89) and dorsal angulation of the dens (90). Occipital dysplasia (i.e., foramen magnum keyhole-like widening) may also be seen (40, 91); however it has been suggested that this is an acquired condition due to overcrowding of the caudal cranial fossa, mechanical pressure from the cerebellum and supraoccipital bone resorption (43). In some cases of CM/SM, a fibrous band caudal to the foramen magnum that compresses the spinal cord and subarachnoid space has been identified (53) and similar atlantoaxial banding has been found in humans (87).

Taken together, the range of craniocervical junction anatomical abnormalities associated with CM/SM highlight the multifactorial nature of this condition in dogs (and humans). How each of these assemblies develops in a coordinated manner during normal embryonic morphogenesis and post-natal growth remains poorly understood.

## Embryological basis of CM

Embryology, as in human CM, provides the means to understanding the developmental inter-relations between the brain, derived from the neuroectoderm, and the skull, derived from mesodermal and neural crest cells (61, 92–94). The multipotent neural crest cells which delaminate from the dorsal neural tube, contribute not only to the peripheral nervous system, but also to the ectomesenchymal precursors that underpins the cranial skeleton and thus the potential to influence irregularities associated with CM. In the following overview, the normal development of the neural and skeletal components are described to give context for identifying where any abnormalities arise that might influence CSF circulation with resultant CM and secondary SM.

### Brain and ventricles

Although the brain and skull form synchronously, their relationship is not necessarily benign, as demonstrated by increased intracranial pressure in craniosynostosis (95, 96). The vertebrate central nervous system arises from a flat neuroectodermal plate to a neuroepithelial tube-like structure which bends at defined closure-initiating points and propagates along the length of the embryo through “zippering”. In the dog, neural tube closure starts around day 16 of gestation and is complete by around day 18 (97, 98). The neural tube is surrounded dorsally by ectoderm, dorsolaterally by paraxial mesoderm and neural crest cells and ventrally by the notochord (99, 100). Failure of neural tube closure causes neural tube defects including craniorachischisis (failure of the initiation of closure), exencephaly (the embryonic precursor of anencephaly, failure of cranial closure) and spina bifida (failure of caudal closure). In humans, spina bifida is commonly associated with CM-II (historically referred to as Arnold Chiari malformation). Several theories have been proposed to explain the biological basis for this association, and has recently been reviewed (101). Canine CM is not classically associated with neural tube closure defects (56).

**Figure 3:**
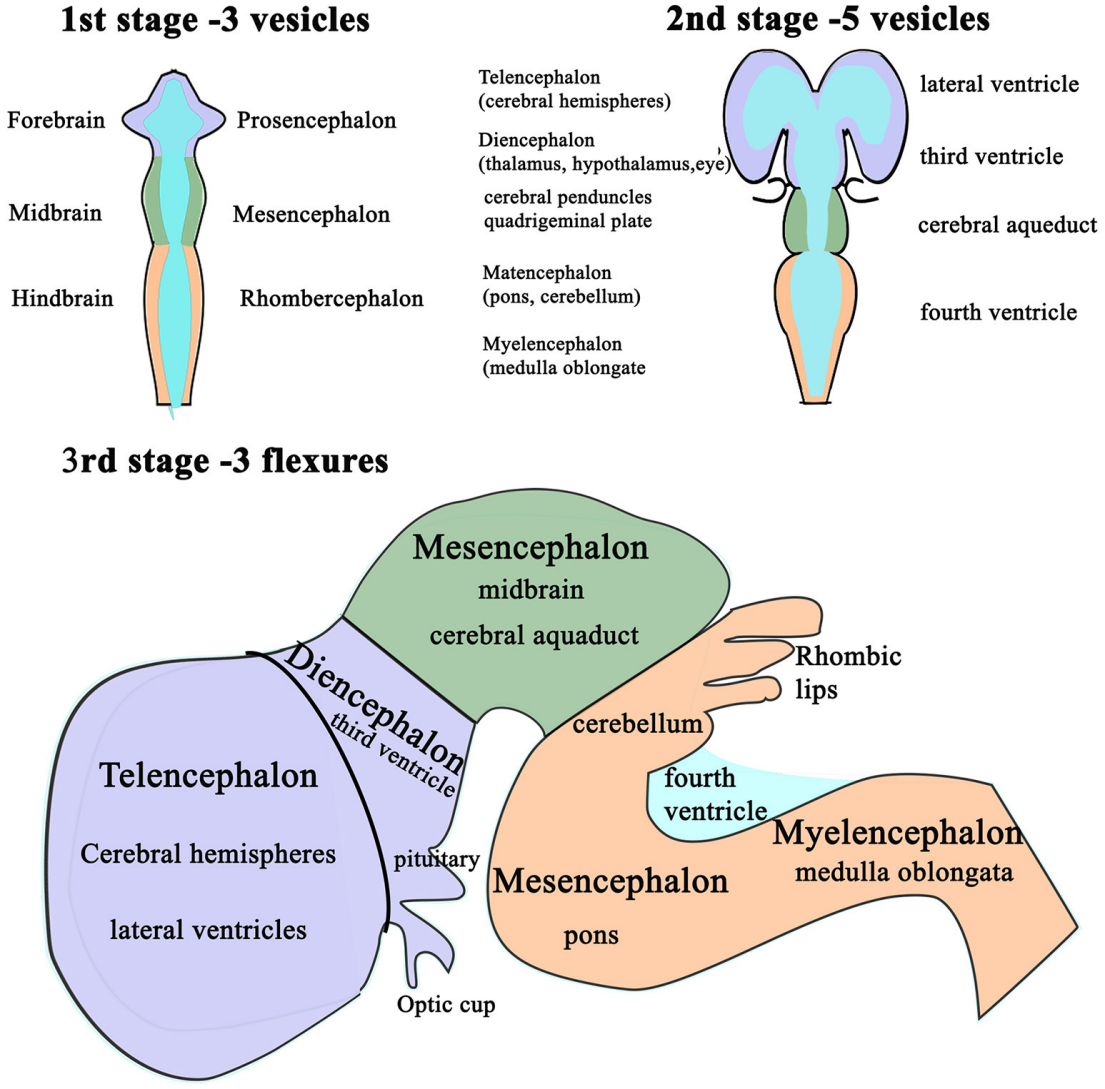
Schematic diagram of the differentiation of vesicles in the developing brain. The three stages in the development of the vesicles within the brain as changes in differential growth and proportions of the forebrain, mid brain and hindbrain (1st and 2nd stages) are modified by flexures that still allow the continuity of the CSF channels within. Stage 3 illustrates the formation of cerebellum and its relationship with the fourth ventricle and the cerebral aqueduct.

Following completion of neural tube closure, hydrostatic pressure is actively increased within its lumen and the cranial neural tube balloons into three distinct segments (102): the presumptive forebrain (prosencephalon), midbrain (mesencephalon) and hindbrain (rhombencephalon) (94, 103, 104). These are punctuated by three primary flexures, two of which convex dorsally; the cephalic flexure at midbrain level and the cervical flexure, at the junction of the hindbrain and the spinal cord, whereas the intervening pontine flexure concaves dorsally (Figure 3).

**Figure 4:**
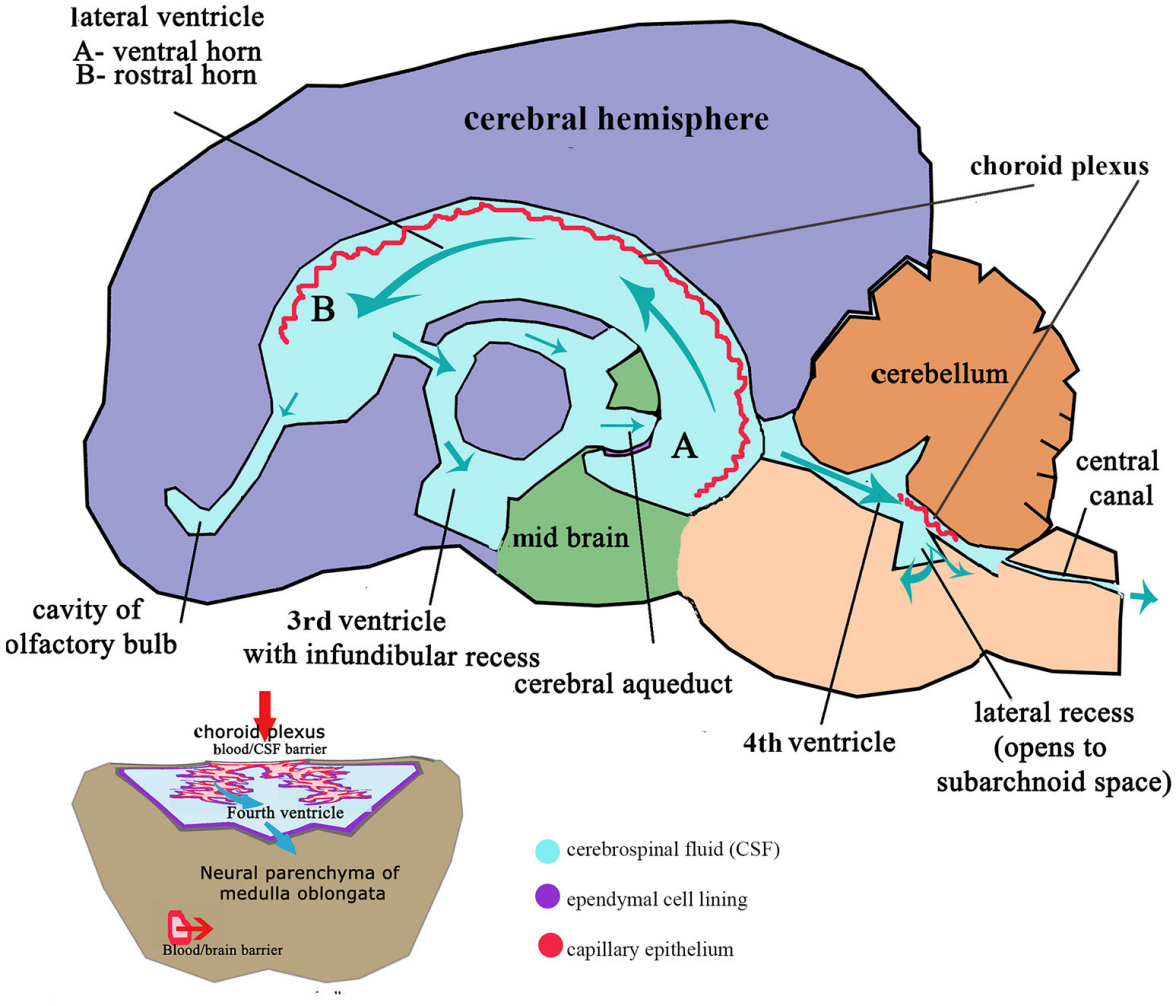
Schematic adult brain with ventricular system with enlarged fourth ventricle inset illustrating formation of CSF. Red arrows indicate movement of filtrate from the venous system into the ventricles at the choroid plexus; Aqua arrows indicate movement of CSF through the ventricles to the subarachnoid space and central canal. Inset: Transverse section of medulla oblongata (myelencephalon) illustrating relationship of the choroid plexus epithelium invaginated into the CSF space of the fourth ventricle and the blood/brain barrier and compliance with CSF drainage.

These flexures, together with differential expansion of the intervening parenchyma, produce the initial shape of the five vesicles (telencephalon, diencephalon, mesencephalon, metacephalon and myelencephalon) and create communicating channels (94); the narrow interventricular foramina (Monroe) which connect the lateral ventricles to the third ventricle, the cerebral or mesencephalic aqueduct (Sylvius) from third to fourth ventricle. The median aperture (Magendie) from fourth to the subarachnoid space via the cisterna magna of primates is not present in dogs (104–106) (Figure 4). The left and right lateral apertures of the fourth ventricle open to the subarachnoid space (61, 104). The ventricles, containing CSF, are lined by ependymal cells which are continuous with the central canal of the spinal cord. Highly vascularized pia mater covered by ependymal epithelium eventually develops into the choroid plexus which invaginates into the lateral, third and fourth ventricles, forming the blood-CSF barrier (Figure 4 inset).

Having established the brain's basic shape, further specification occurs. The rhombencephalon patterns into eight distinct segments, referred to as rhombomeres, which form distal to the cephalic flexure under the control of Hox gene transcription factors. Each rhombomere gives rise to a distinct hindbrain segment with its own set of ganglia and nerves. The cerebellum starts as a proliferation of the rhombic lips (Figure 4), which expand medially through the roof plate of the fourth ventricle, fusing in the midline. As the pontine flexure deepens, the enlarged metencephalon becomes folded against the dorsal lamina of the medulla and developing choroid plexus (94). Neuroepithelial cells in the rhombic lips differentiate along various lineages. It is pertinent to note this later growth of the cerebellum in the context of its deformation in CM. In the human fetus, it has been shown that cerebellar growth commences later than the cerebral hemispheres, whereas the growth of the bony posterior fossa appears to precede cerebellar growth (107). It is credible, therefore, that any reduction in the proportions of the bony caudal fossa does not inhibit growth of the cerebellum which would develop “normally” and become impacted or herniated within the reduced caudal fossa volume. Indeed, it has been demonstrated that CKCS have an enlarged cerebellum relative to the volume of the entire brain (38).

The traditional model of CSF hydrodynamics suggests the majority of CSF drains from the subarachnoid space surrounding the brain mostly via arachnoid villi in the dorsal sagittal sinus and distal recesses of meningeal sheaths surrounding nerve roots, especially the optic and olfactory nerves. The choroid plexus (Figure 4 inset) provides a large surface area for solute exchange and CSF flow as a consequence of water filtration between the capillaries and interstitial fluid. However, the classic CSF physiology, that this organ represents a powerful biological pump, is now challenged in favor of CSF exchange that is constant and present everywhere in the system (77, 108). There is increasing evidence of the important role of extracranial lymphatic vessels plays in CSF dynamics (79, 84, 109–111). Brachycephalic breeds have a restricted rostral dorsal sagittal venous sinus compared to dolichocephalic and mesaticephalic dogs (63) and the additional reduced olfactory lobes might adversely affect CSF drainage and thereby increase risk of CM/SM. Disturbance of the CSF circulation in a critical stage of development can lead to a range of pathologies such as congenital hydrocephalus and ventriculomegaly associated with CM/SM (104, 112).

Thus, expansion and early growth of the developing brain, initially without surrounding skeletal components, may be subsequently be subjected to mechanical forces by skull formation that can impinge on late pre-natal and early post-natal morphogenesis depending on the elastic properties of the brain parenchyma (113). It is reasonable to propose that this and selected impairment and dysfunction of CSF flow, may, over a period of time, result in late onset SM. Indeed, recent research suggests that brain lymphatic system and its regulation are influenced by physiological conditions such as aging, genetic phenotypes, heart and respiration and it may have a role as potential therapeutic targets in the treatment of neurological diseases and pain (114).

### Skull

**Figure 5:**
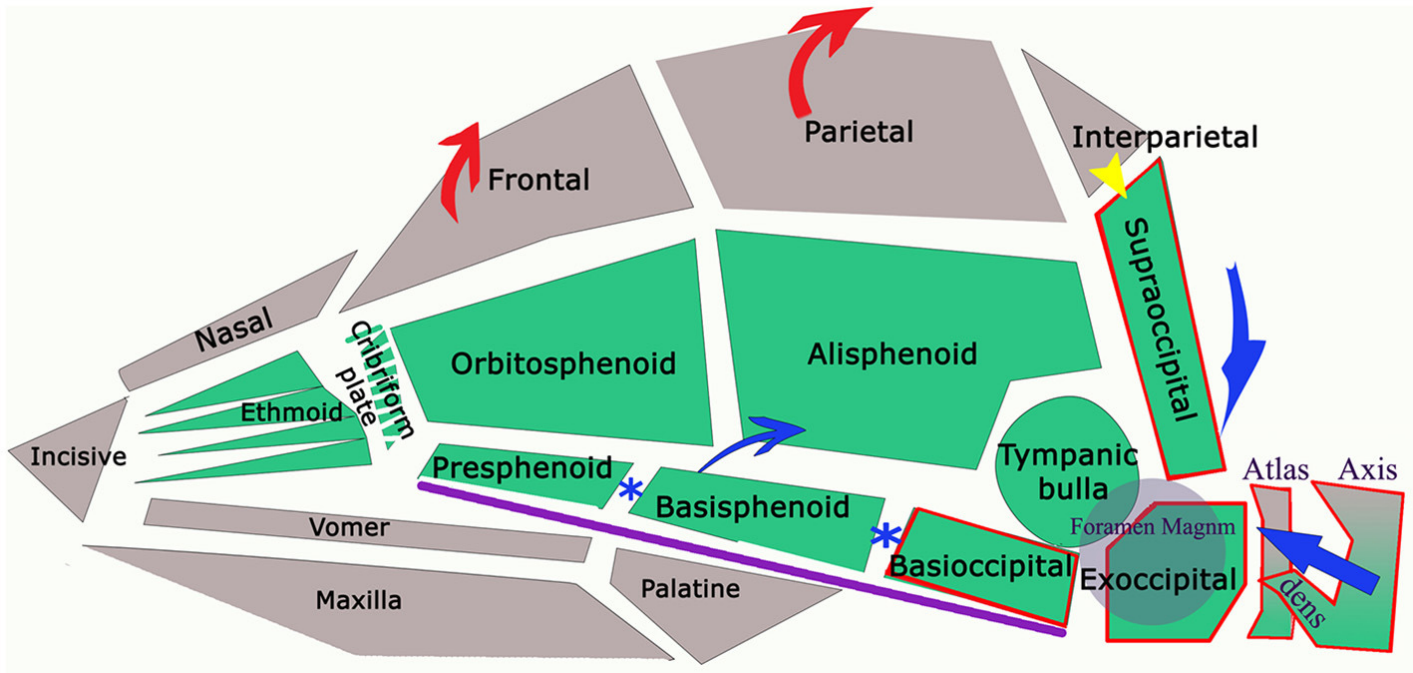
Diagram of the bones referred in text (lower jaw not illustrated) with arrows to indicate anatomical changes in dogs with CM. The green colored bones are endochondral in origin (neurocranium of skull and notochord in axial skeleton). Calvaria and facial bones have membranous ossification (viscerocranium). Sclerotomal occipital region bones (i.e., craniocervical junction) indicated by red border. Purple line indicates the skull base. In dog and man, interparietal and supraoccipital fuse (yellow arrow). The reduced volume of the caudal fossa in CM is indicated by the blue arrows: reduction of the supraoccipital and dorsal displacement of atlas and axis; premature closure of synchondroses of bones in skull base (blue*) shortening the skull base and compensatory increase in height of the parietal bone and frontal bone (red arrows).

The canine skull, as in other vertebrates, is a product of its evolutionary origins whereby the loss of lateral walls of the neurocranium in both birds and mammals enabled the brain to expand into the dermal skull roof (115). Four skull components can be described [Figure 5, based on (115)].

1. Neurocranium: trough-like skull base which has a primitive cartilaginous forerunner (chondrocranium) supporting the brain and sensory organs.
2. Viscerocranium (splanchnocranium or orognathofacial complex): modified cartilaginous supports for the gill arches of early aquatic vertebrates associated with feeding but also part of the senses. In later evolutionary vertebrates these features contribute to the jaws, hyoid and inner ear bones (61, 116).
3. Dermatocranium or cranial vault (calvaria): dermal or intramembranously-ossified bones which encase the telencephalon and the nose which originated from the bony head armor without a cartilaginous precursor.
4. Sclerotomal occipital region: incorporation of the occipital vertebrae into the skull, supported by the annexation of the cranial part of the spinal cord into the brain, together with the first 2 spinal nerves as cranial nerves X1 and X11 (117).

Characteristic canine head conformations are assumed to arise in part from breed-specific patterns in cranial growth and suture closure. The timing of suture closure between the ten bones comprising the cranium (six unpaired and four paired) is genetically predetermined (discussed further below). CM-associated features include a reduction in the length of the cranial base to include the basioccipital and basisphenoid and volume of the caudal fossa, smaller and ventrally rotated olfactory bulb, rosto-dorsal variations in the clival angle (cranial base angulation between the ethmoidal plane and spine) and increased proximity and more acute angulation of the atlas and dens (11).

Prenatally, skull growth is uneven, reflecting and accommodating the developing brain (118). When intracranial pressures become excessive, as in hydrocephalus, both cortical plates of the calvaria become thinned and grossly expanded (119, 120). Conversely, reduced brain expansion is associated with small calvaria, such as in microcephalics (121). External functional forces acting on the calvarium, such as from muscles of mastication, add to calvarial dimensions, but not to the intracranial capacity (118). Mechanical stresses are absorbed at the sutures by cellular proliferation and fiber formation which creates immovable bone joints (synarthroses) (122). Their size, type and location are genetically determined and closure occurs in a defined pattern in relation to the brain (123).

Expansion of the cranial base occurs by primary growth of the cartilage and by expansion at the synchondroses i.e., at the suture lines (123) and craniosynostosis (premature fusion) is recognized as a key component of skull abnormalities including CM/SM (124, 125). The median elements of the basicranium (the basioccipital, basisphenoid and paired ossifications for the presphenoids and ethmoid bones) fuse with their lateral component wing (orbitosphenoids and alisphenoids) to form the trough-like sphenoidal complex. Fusion between these two sphenoids is never complete in the dog (61). A recent study explored breed-specific patterns of cranial suture and synchondrosis closure in relation to airorhynchy (preaxial angle) in domestic dogs. By comparing the closure of 18 sutures in domestic dogs with the wolf, they identified a correlation between patterns of closure which appear to underlie variations in skull shape. For example, the basisphenoid-preshenoid synchondrosis is significantly positively correlated to the prebasial angle and the sutures of the nose and palate. Brachycephalic breeds such as the Bulldog had significantly higher closure scores than non-brachycephalic breeds and the wolf (126). In addition to regulation of cranial expansion through suture closure, the rostral and caudal sections of the skull (divided by the hypophyseal fossa or sella turcica) increase their length at different rates and at different times, with the sphenoid and basioccipital bones developing more slowly. The cranial base is angled at the level of the hypophyseal fossa where the rostral prechordal and caudal chordal parts meet. A study of neonatal CKCS (127) found that the lambdoid suture closed earlier the CKCS when compared to Beagles. Premature closure of the lambdoid suture is associated with reduction in the posterior fossa volume in human CM-1 (128).

The spheno-occipital synchondrosis makes a significant contribution to growth postnatally because chondral growth of the synchrondroses continues until the bones are ossified (103). Premature fusion of the joint between the basisphenoid and the basioccipital (spheno-occipital synchondrosis) and between the basisphenoid and presphenoid (intersphenoidal synchondrosis) shortens the basicranial axis (Figure 5). However this allows the lower jaw to continue to grow unopposed rostrally and arch dorsally in brachycephalic dogs, as typical of such breeds as the Bulldog and Griffon Bruxellois (61, 67, 129). The spheno-occipital synchondrosis has been shown to ossify earlier in CKCS compared to other brachycephalic dogs, which in turn ossified earlier than mesaticephalic dogs (130). Furthermore, complete removal of the spheno-occipital synchondrosis in experimental rats drastically changes skull growth resulting in observable differences in the angulation of the skull base, an increased curvature of the cranial roof and a marked forward displacement of the occipital condyles reminiscent of CM skull conformation. Other changes included a ventral and forward rotation of the plane of the foramen magnum (131). Rostral dorsal angulation between the skull base and hard palate (129) and ventral rotation of the brain (60) is typical of brachycephalic breeds.

### Craniocervical junction

At the craniocervical junction, the four endochondral occipital bones that surround the foramen magnum do not have the same embryological origins in the mammalian skull (Figure 5) (132). The basioccipital bone of the chondrocranium is the first of the bones in the skull base to ossify and the two lateral exoccipitals follow the changing contours of the developing brain (98). The supraoccipital bone, forming the dorsal border of the foramen magnum, is derived from cephalic paraxial mesoderm (92, 133) and its development is influenced by the evolution of the interparietal bone, which becomes incorporated with the supraoccipital in many mammalian species including man and the dog. As part of an investigation into the development of CM in the CKCS, (127) studied the histology of the supraoccipital bone in neonatal dogs and identified two parts: superior-membranous and inferior cartilaginous. A sinus was identified between the two parts of the bone, located on the internal surface of the occipital protuberance implying that the two parts ossified and fused at birth. In humans, the interparietal bone begins to fuse before birth and closure is completed between the second and fourth year (134). Sometimes the bones can remain unfused in the adult dog, more apparent internally or as the external sagittal crest (61). The nuchal crest separates the dorsal and caudal divisions of the skull and the external occipital protuberance forms a ridge or crest which is poorly developed in some dogs, e.g., Griffon Bruxellois (18, 67) but particularly in dogs with CM/SM (32).

Occipital hypoplasia has been a key identifying features of SM secondary to CM (135) and results in a reduced volume of the caudal fossa which plays a significant role in the pathogenesis of SM. Furthermore, histology of neo-natal CKCS showed marked apoptosis of supraoccipital chondrocytes, which would result in decreased growth potential (127). Histomorphometric analysis confirmed an irregular and concave bone with reduced numbers of trabeculae (bony spicules which form a meshwork of intercommunicating spaces), compared to age-matched controls (different breeds). In other words, the quality of the supraoccipital bone in the CKCS fetus was diminished. Pathological changes with a lower cellularity were also identified in the single sample of adult supraoccipital bone (40).

**Figure 6:**
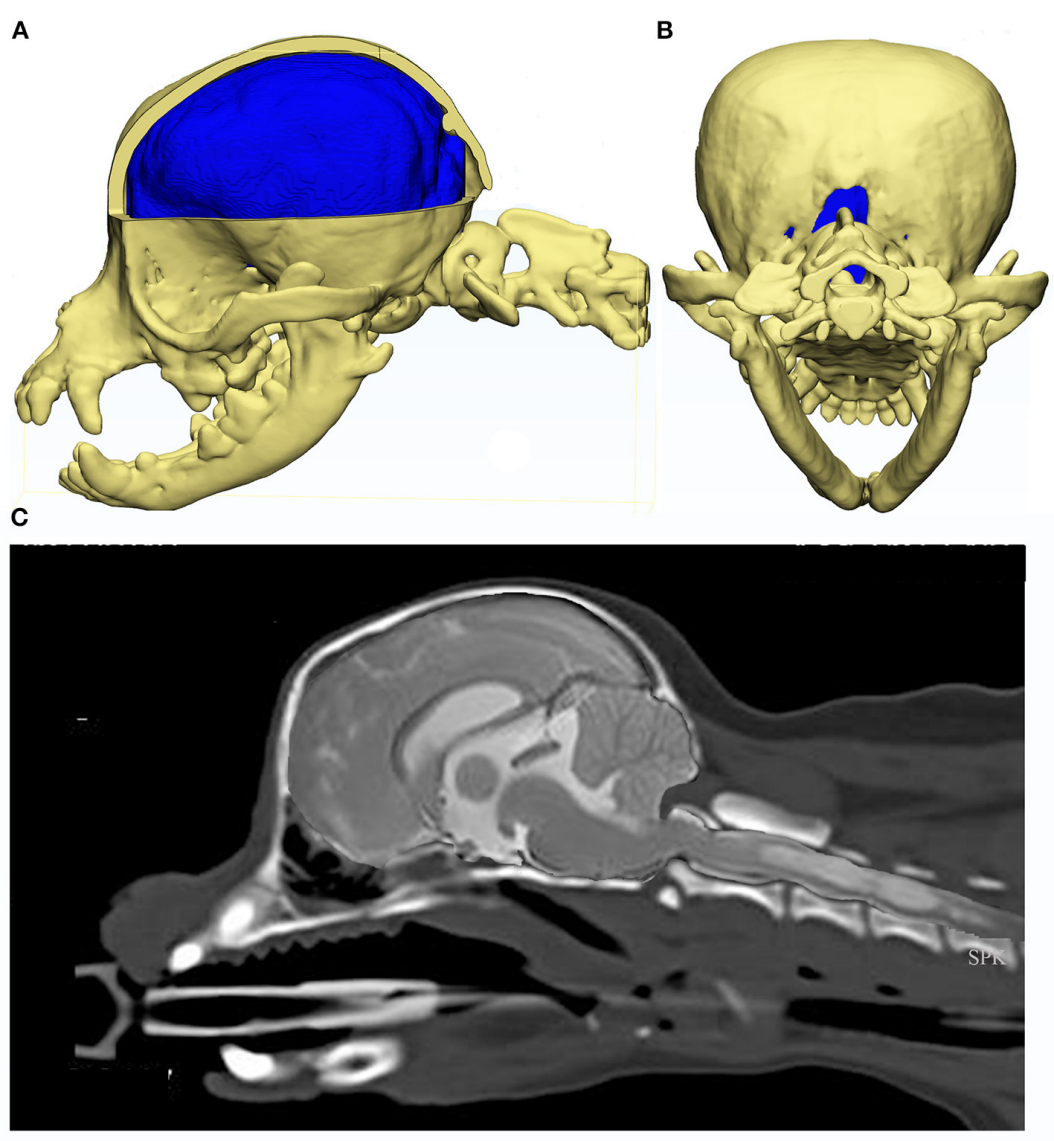
Constructed images using CT and MRI of the skull, brain and craniocervical junction of a Chihuahua with CM and SM. MRI and CT of a 5 year old female Chihuahua with CM and SM are used to construct images to illustrate the complexity of the skull, craniocervical junction and neuroparenchymal malformation. Top row: 3d models (Courtesy of Dr. Cameron Black, Clinical Research Officer, Fitzpatrick Referrals Ltd.) **(A)** cerebellum is invaginated under the occipital lobes with little space available for this organ. Much of the atlas is rostral to the level of the occipital crest. **(B)** caudal view of the skull with the supra-occipital bone missing either because it did not form in the first instance or was resorbed due to pressure necrosis. Bottom row: **(C)** composite of midsagittal brain and cervical MRI and CT with the short skull base and complex craniocervical junction abnormality with overall reduction of volume, loss of the cisterna magnum and medullary elevation over a dorsally angled den.

Rusbridge established a coexistence of occipital dysplasia and hypoplasia with SM in CKCS and demonstrated that it was not possible to differentiate by MRI either condition with herniation of the cerebellum (40). Post mortem findings identified a tough membrane that covered the keyhole defect and suggested that this tissue was more compliant than bone and offered less resistance to CSF flow dynamics during the cardiac cycle. It was postulated that this might also delay onset of painful clinical signs associated with CM (40). However it was later suggested that the bone defect was not congenital but acquired and bone was resorbed due to pressure from caudal fossa overcrowding. Indeed, Driver et al. showed that the height of the foramen magnum in CKCS increases with time (43), i.e., suggesting a dynamic loss of the supraoccipital bone, and may be a marker of disease progression (45). Figure 6 provides an example of a Chihuahua with the supraoccipital bone is completely lost which might be attributed to pressure necrosis, with the dens dorsally angled. The atlas is rostral to the occipital crest but not actually overlapping, as nothing to overlap in this individual. Atlanto-occipital overlap can occur with and without SM in this breed (16, 32, 85).

The overall complexity of the embryology of the skull and craniocervical junction has resulted in range of abnormities which results in a mismatch between the skull, craniocervical junction and neuroparenchyma that might compromise CSF circulation. The association of CM with craniofacial abnormalities that are paraxial mesoderm in origin and involve craniosynostosis is well documented in humans (9, 134). Unfortunately, the desire and popularity to select and breed dogs that produce characteristic facial and breed specific features has resulted in breed-specific variations in suture/synchondrosis fusion which interfere with the natural morphogenetic processes of growth, thus predisposing dogs to CM.

## Genetics of CM

Embryological morphogenesis as well as the subsequent rate/extent of growth are under genetic control. Polygenic contributions of complex disorders are well-established, with each variant typically explaining a small proportion of the disease phenotype or risk. Canine CM is known to have a strong heritable component (22, 136, 137). Breeding away from SM has been shown to be effective (28, 138) and attempts have been made to elucidate the inheritance of brachycephaly using mixed breeding with a mesaticephalic dog (139). Research at Montreal and McGill Universities with principal investigator Dr Z Kibar investigating human genetics of CM has made use of canine databases from worldwide collections (140). CM is ubiquitous in the CKCS and consequently there were insufficient control dogs without CM/SM available so this breed was used to investigate SM secondary to CM. The Griffon Bruxellois breed was used to investigate CM. In the CKCS, an initial genome wide linkage studies identified a novel locus for SM associated with CM and a haplotype that infers protection against SM (141). A more recent study involving 526 dogs, identified two loci on *Canis familiaris* Autosomes (CFA) 22 and CFA26 which were associated with the syrinx maximum transverse diameter. A wide syrinx diameter was linked to reduced volume of the caudal cranial fossa illustrated by the phenotype in Figure 2 which was used for genotyping (136). A quantitative trait locus (QTL) approach was adopted in the Griffon Bruxellois and analyses identified associated single nucleotide polymorphisms (SNPs) on 5 CFAs: CFA2, CFA9, CFA12, CFA14 and CFA24. A reconstructed haplotype of 0.53 Mb on CFA2 strongly associated to the height of the cranial fossa and CFA14 was associated with both the height of the rostral part of the caudal cranial fossa and the height of the brain. The CFA2 QTL harbors the Sall-1 gene which is an excellent candidate since its orthologue in humans is mutated in Townes-Brocks syndrome with known association to CM- I (137).

Given the relative rarity of the condition, human CM-I studies have largely relied on analysis of candidate genes with known roles in embryonic development [e.g., (142)]. A recent whole exome sequencing study analyzing two Italian pedigrees identified potentially causative mutations in three genes: Dickkopf-like (DKK)1, LDL receptor-related protein (LRP)4, and bone morphogenetic protein (BMP)4 (143). Both DKK1 and LRP4 are components of the potently-osteogenic canonical Wnt signaling pathway and both have antagonistic effects on this pathway. DKK1 acts as a secreted antagonist of Wnt-ligands, whereas LRP4 potentiates the effects of another secreted Wnt antagonist, sclerostin (144). Intriguingly, BMP signaling is also potently osteogenic, a property which is used clinically in fracture repair.

A missense mutation in another BMP ligand, BMP3, has previously been associated with variations in skull shape in dogs (145). This mutation was almost exclusively found in extreme brachycephalic breeds. A more recent genome wide association study found that the genetic polymorphisms with the largest effect size on neurocranium centroid size (the square root of the sum of squared distances of landmarks from the centroid: a measure which takes into account influences of allometry from viscerocranium shape variation) were in SMAD2, which acts as a BMP effector in certain cell types (146). In addition, an intronic transposon which reduces expression of the gene SMOC2 was found to explain 36% of the variation in face length between breeds, and skulls from *Smoc2*^−/−^ mice were found to be widened mediolaterally and shortened rostrocaudally (146). SMOC2 is a secreted matricellular protein involved in cell adhesion and migration, but it has also been reported to promote BMP target gene expression in zebrafish (147).

Genetic contributions are likely to be polygenic, involving those that influence cranial base length and synchondrosis closure time, supraoccipital and intraparietal bone development, size of the cerebellum, morphology of the craniocervical junction and possibly factors that affect CSF production and absorption. Environmental factors, epigenetic factors and/or other genetic modifiers may also be responsible for the final phenotype of CM/SM perhaps acting through the blood/brain/CSF barrier associated with BOCCS. Taken together, these studies strongly support the involvement of embryonically-active pro-osteogenic signaling pathways in the establishment of cranial structure, potentially supporting the theory that compression of the neuroepithelial brain by predominantly mesodermally-derived skeletal components is caused by mesodermal insufficiency (148). This may have an evolutionary basis: variable tandem repeat sequences in the master regulator of the bone-forming osteoblast lineage runt-related transcription factor (RUNX)2 is associated with face length across carnivore species as well as specifically within dogs (149). Although the majority of studies reported to date have focused on facial length rather than craniofacial index, the large effect sizes identified raise the possibility that similar genetic pathways may influence risk of developing CM. Robust phenotyping is essential for genetic investigation and further delineation of the genetic basis of canine CM/SM and potentially identify high-risk individuals at a very early age in order to allow selective monitoring or even the development of prophylactic targeted therapies.

## Conclusion and future developments

This review reflects the key morphogenetic processes involved in CM/SM and how they relate to the most recent research findings for these conditions with respect to brachycephaly, craniocervical junction abnormalities and diagnosis but highlighting the need for further investigation.

Understanding the developmental origins of complex three dimensional structures of the brain, skull, craniocervical junction and spinal cord with associated ventricles and CSF channels, is crucial to understanding canine CM and ultimately the pathogenesis of SM. The molecular genetics that underlay CM/SM can provide a powerful tool to elucidate such an understanding. However, the challenge is finding appropriate genetic markers that can achieve this, which is confounded by not having readily quantifiable pathogenic phenotypic features to study. The importance of accurate phenotyping has been fundamental for genetic studies of CM, but it is also the key for accurate diagnosis (14, 15). The complexity of the existing morphometrics involved in the current phenotypes make them impractical for everyday use and a machine learning technique for diagnosis that removes human bias has been suggested (150). Pilot studies in this technology has identified biomarkers in structures such as the soft palate not previously associated with these conditions that when investigated may give further insight into conformation change. Mathematical modeling has also been applied to the dynamics associated with CSF flow which might be used to predict the outcome following different surgical approaches (151). The challenge of quantifying and interpreting intermittent and variable clinical signs for a complex trait such as CM/SM cannot be overstated. More investigation is required to defining the pathology of symptomatic CM and its relationship with brachycephaly and miniaturization. This priority for future research can make a considerable and direct impact on dog welfare.

The genetic investigation for canine CM/SM has been carried out in conjunction with human studies and it reaffirms how “Man and his Dog” have an enduring partnership. The progress made has been with the whole-hearted co-operation of breeders and pet owners supporting human sufferers of CM/SM under the umbrella “One Health.”

## Author contributions

CR: Concept; SK: Original draft and graphics; GG and CR: Editing and reviewing draft.

### Conflict of interest statement

The authors declare that the research was conducted in the absence of any commercial or financial relationships that could be construed as a potential conflict of interest.
